# Effect of inpatient antibiotic treatment among older adults with delirium found with a positive urinalysis: a health record review

**DOI:** 10.1186/s12877-022-03549-8

**Published:** 2022-11-29

**Authors:** Pil Joo, Lars Grant, Tim Ramsay, Caroline Nott, Rosemary Zvonar, Jason Jia, Krishan Yadav, Eisi Mollanji, William He, Debra Eagles

**Affiliations:** 1 Department of Family Medicine, University of Ottawa, Ottawa, Ontario Canada; 2 Ottawa Hospital Research Institute, 1053 Carling Ave, ON K1Y 4E9 Ottawa, Canada; 3 McGill University, Montreal, Quebec, Canada; 4 School of Epidemiology and Public Health, University of Ottawa, Ottawa, Ontario Canada; 5 Department of Medicine, University of Ottawa, Ottawa, Ontario Canada; 6 The Ottawa Hospital Pharmacy Department, Ottawa, Ontario Canada; 7 Department of Emergency Medicine, University of Ottawa, Ottawa, Ontario Canada; 8 Faculty of Medicine, University of Ottawa, Ottawa, Ontario Canada

**Keywords:** Delirium, Asymptomatic bacteriuria, ASB, UTI, Choosing wisely

## Abstract

**Background:**

Among older adults with delirium and positive urinalysis, antibiotic treatment for urinary tract infection is common practice, but unsupported by literature or guidelines. We sought to: i) determine the rate of antibiotic treatment and the proportion of asymptomatic patients (other than delirium) in this patient population, and ii) examine the effect of antibiotic treatment on delirium resolution and adverse outcomes.

**Methods:**

A health record review was conducted at a tertiary academic centre from January to December 2020. Inclusion criteria were age ≥ 65, positive delirium screening assessment, positive urinalysis, and admission to general medical units. Outcomes included rates of antibiotic treatment, delirium on day 7 of admission, and 30-day adverse outcomes. We compared delirium and adverse outcome rates in antibiotic-treated vs. non-treated groups. We conducted subgroup analyses among asymptomatic patients.

**Results:**

We included 150 patients (57% female, mean age 85.4 years). Antibiotics were given to 86%. The asymptomatic subgroup (delirium without urinary symptoms or fever) comprised 38% and antibiotic treatment rate in this subgroup was 68%. There was no significant difference in delirium rate on day 7 between antibiotic-treated vs. non-treated groups, (entire cohort RR 0.94 [0.41–2.16] and asymptomatic subgroup RR 0.69 [0.22–2.15]) or in 30-day adverse outcomes.

**Conclusions:**

Older adults with delirium and positive urinalysis in general medical inpatient units were frequently treated with antibiotics – often despite the absence of urinary or other infectious symptoms. We failed to find evidence that antibiotic treatment in this population is associated with delirium resolution on day 7 of admission.

**Supplementary Information:**

The online version contains supplementary material available at 10.1186/s12877-022-03549-8.

## Introduction

Delirium is a common finding in older adults, representing 10% of emergency department (ED) visits and occurs in 29–64% among those hospitalized [1, 2]. In such older adults with new or worsening confusion, urinary tract infection (UTI) is often sought and treated with antibiotics, even in the absence of localizing genitourinary symptoms or signs [3–6]. However, asymptomatic bacteriuria (ASB) is also common in older adults, with a prevalence as high as 25–50% [7, 8]. Delirium and ASB are both more common among older adults with baseline cognitive impairment [9]. Studies have shown little benefit with antibiotic treatment of ASB in patients without delirium, while reiterating its harms [8]. Nevertheless, there is a paucity of literature to guide clinicians managing older adults with delirium and positive urinalysis but without other findings pointing to UTI, especially in inpatient settings. Systematic reviews have found that studies investigating the relationship between UTI/ASB and delirium were methodologically flawed, and the causal relationship between these condition remains elusive [4, 10, 11]. Many studies included in these systematic reviews had no specific definition of UTI other than as noted in the medical records, or included delirium as part of the definition. On the other hand, non-specific symptoms such as altered mental status, lethargy or malaise did not appear to increase the probability of bacterial infection in one study [12]. Recent guidelines from the Infectious Disease Society of America and the Association of Medical Microbiology and Infectious Disease of Canada recommend assessment for causes other than UTI in cognitively or functionally impaired older patients with bacteriuria and delirium [8, 13].

Despite these recommendations, treatment of suspected UTI based on positive urinalysis in confused older patients is a common practice even when localizing UTI symptoms or systemic signs such as fever are absent [3, 5]. Ideally, UTI and ASB should be diagnosed in the context of positive urine culture. However, given the multi-day delay to obtain urine culture results, urinalysis is often used in practice as a surrogate marker to make decisions for antibiotic treatment, despite its inherent limitations in specificity and sensitivity [14]. Despite this common practice, whether such practice has any benefit in improving the rate of delirium resolution is unknown.

For this reason, we wished to gather data to enable design of future clinical trials as called for by the previous studies [4, 5, 15]. We conducted a health record review of inpatient older adults with delirium and positive urinalysis. We sought to examine how many patients were treated with antibiotics among two subgroups: the group with infectious symptoms or signs beyond delirium and positive urinalysis (potential UTI, pneumonia, etc.), and the group with none (potential ASB). We then attempted to examine the association between antibiotic treatment and delirium resolution, and adverse outcomes, including mortality and intensive care unit (ICU) admission.

## Methods

### Study design and setting

This health record review examined older adults admitted to general medical units of The Ottawa Hospital, an academic tertiary-care hospital in Ottawa, Ontario. We followed STROBE guidelines for reporting observational studies [14]. The study was approved by the Ottawa Health Science Network Research Ethics Board (#20190271-01H).

### Patient population

The Ottawa Hospital’s Data Warehouse identified charts for patients meeting the inclusion criteria of: i) aged 65 or older, ii) admitted from ED to either Family Medicine or General Internal Medicine services, iii) had a positive delirium screening with the Brief Confusion Assessment Method (bCAM, described below) within 36 hours of admission, and iv) had a positive urinalysis within 36 hours of admission. We defined urinalysis to be positive if a point of care or laboratory urinalysis showed trace or greater amounts of leukocyte esterase or positive nitrites [16]. Patients with an indwelling urinary catheter, including a suprapubic catheter, nephrostomy tube, or urologic stent, were excluded. Patients who were admitted repeatedly within the study period were included only once, on their first admission*.*

### Outcome measures

The primary outcome was the proportion of patients that were treated with antibiotics, including those with indication for antibiotics and those without indication for antibiotics other than the inclusion criteria of delirium and positive urinalysis. Indications for antibiotics were UTI symptoms, reported or measured fever, or other infectious diagnoses that may warrant antibiotic treatment. (For consistency, we used the term “asymptomatic” if a patient did not have UTI symptoms, fever or other indications for antibiotics.) UTI symptoms were defined as frequency, urgency, dysuria, suprapubic tenderness or costovertebral angle/flank tenderness (adapted from the Loeb criteria) [17]. Appendix 1 lists all the diagnoses we encountered in this study and categorizes them as infectious diagnoses potentially requiring antibiotic treatment or otherwise.

We also sought the association between antibiotic treatment and delirium on the seventh day of admission and patient-oriented adverse outcomes within 30 days of admission: mortality, *Clostridioides difficile* infection, ICU admission and alternate level of care (ALC) designation.

The presence of delirium was defined by a positive Brief Confusion Assessment Method (bCAM). The bCAM is a validated diagnostic tool for delirium with 78% sensitivity and 97% specificity when performed by a non-physician [18]. It consists of four rapidly administered questions, resulting in dichotomous diagnosis of delirium or non-delirium. It is administered routinely by nursing staff on inpatient units of The Ottawa Hospital. Typically, a bCAM assessment is initiated when the patient is transferred to an inpatient unit from ED, but this transfer can be delayed for several days. For this reason, patients with a positive bCAM documented at any time between ED assessment to the first 36 hours of admission were considered to have delirium at admission and included in the study. We considered a patient to have delirium on day 7 of their admission if there was one or more positive bCAM results on that day. The seventh day of admission was chosen based on a recent survey which showed that 96% of physicians answering the survey expected improvement of delirium within seven days if antibiotic treatment was effective [3]. Patients were considered part of the antibiotic group if they received any antibiotics at any time between ED presentation and the first 36 hours of admission.

Alternate level of care (ALC) designation is given to patients in Ontario whose active inpatient medical treatment is completed but are still unable to be discharged to a pre-admission living arrangement and represent worsening functional outcome.

### Data collection

We followed robust methodological standards for chart reviews including abstractor training, definition of variables a priori, use of a standardized case record form, development of a code book, and evaluation of inter-rater reliability [19].

An electronic standardized data abstraction form was created on the REDCap platform and was further refined using encounters in December 2019 [20, 21]. Study data were collected from January 1st to December 16th of 2020. A code book for the data abstraction form was used to ensure consistency. Three trained researchers (EM, WH and JJ) independently extracted variables of interest. When any data were unclear, the health record was reviewed by a fourth researcher (PJ) and decisions were made by consensus at periodic meetings*.* A random sample of 49 (33%) included charts were abstracted independently by two researchers (EM and WH) to assess inter-rater agreement.

### Data analysis

As a primarily descriptive study, without prior studies to estimate the effect size, we arbitrarily chose to examine 150 consecutive cases meeting inclusion and exclusion criteria.

The data were analyzed in R [22]. Continuous variables were summarized with means and standard deviation or median and interquartile range, as appropriate: means for roughly symmetric distributions and medians if the distribution was obviously skewed. Categorical variables were presented with proportions and compared with relative risk with 95% confidence interval which were calculated with epiR library [23]. Cohen’s kappa statistics were calculated for inter-rater agreement. Given the exploratory nature of this observational study, no formal hypothesis tests were conducted and no *p*-values are provided.

We compared the bCAM positivity rate on the seventh day of admission and 30-day adverse outcome rates between the cohort given antibiotics against those who were not given. We also performed a prespecified subgroup analysis of the asymptomatic cohort (as defined in the “Outcome measures” section above).

## Results

**Fig. 1:**
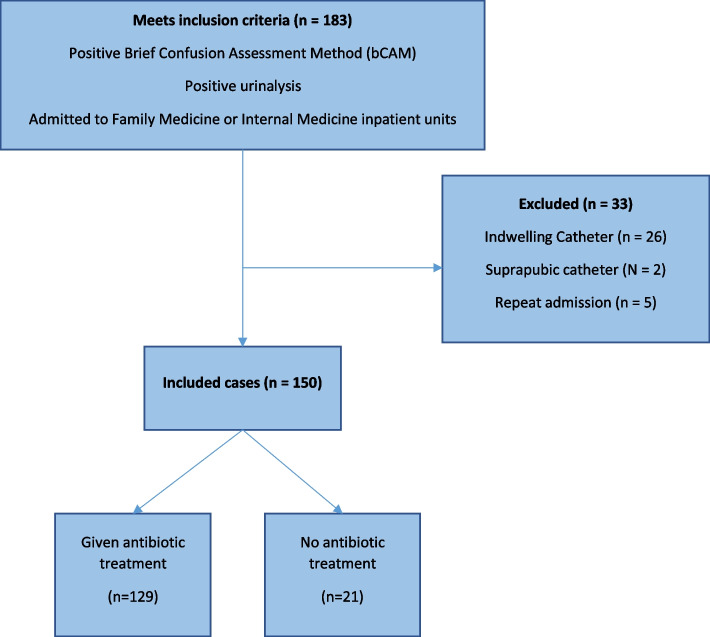
CONSORT study flow diagram

We screened 183 cases meeting the inclusion criteria and 33 cases were excluded, leaving 150 cases that were fully abstracted and analyzed (Fig. 1).

Baseline characteristics are presented in Table 1. A large proportion of patients (86%) were given antibiotics. Both the antibiotic-treated and non-treated groups were similar in most demographic factors and comorbidities. However, we noted certain differences such as the pre-admission living arrangement (independent living in 43% of antibiotic-treated cohort vs. 67% of non-antibiotic-treated cohort) and the admission service (family medicine service admission in 7.8% of antibiotic-treated cohort vs. 29% of non-antibiotic-treated cohort). We also noted that 80% (56 out of 70) of independent patients received antibiotic treatment but this number was higher at 91% (39 out of 43) and 94% (32 out of 34) among patients from retirement home and long-term care, respectively.

**Table 1:** Demographic characteristics and clinical features of included charts

	All patients (*N* = 150)	Given antibiotics (*N* = 129)	Not given antibiotics (*N* = 21)
Age (mean, [SD]) years	85.4 [7.2]	85.5 [7.2]	84.9 [7.6]
Female	85 (57%)	75 (58%)	10 (48%)
Living arrangement			
Independent	70 (47%)	56 (43%)	14 (67%)
Retirement home	43 (29%)	39 (30%)	4 (19%)
Long-term care	34 (23%)	32 (25%)	2 (10%)
Unknown/unclear	3 (2%)	2 (2%)	1 (5%)
Arrival by ambulance	112 (75%)	96 (74%)	16 (76%)
Admission service			
Internal medicine	134 (89%)	119 (92%)	15 (71%)
Family medicine	16 (11%)	10 (8%)	6 (29%)
Length of stay (median, [IQR]) days	18 [11, 29]	18 [12, 29]	14 [9, 33]
Comorbidities			
Diabetes	38 (25%)	34 (26%)	4 (19%)
CAD	22 (15%)	20 (16%)	2 (10%)
CHF	26 (17%)	22 (17%)	4 (19%)
COPD/Asthma	29 (19%)	26 (20%)	3 (14%)
CKD	23 (15%)	18 (14%)	5 (24%)
CVA	26 (17%)	22 (17%)	4 (19%)
Dementia	77 (51%)	64 (50%)	13 (62%)
Cancer	30 (20%)	27 (20%)	3 (14%)
Pelvic prolapse	6 (4%)	6 (5%)	0
UTI within last 6 months	23 (15%)	21 (16%)	2 (10%)
Abx within last 6 months	76 (51%)	69 (54%)	7 (33%)
Antibiotic given	129 (86%)		

*SD*  Standard Deviation; *IQR*  Interquartile Range; *CAD*  Coronary Artery Disease; *CHF*  Congestive Heart Failure; *COPD*  Chronic Obstructive Pulmonary Disease; *CKD*  Chronic Kidney Disease; *CVA*  Cerebrovascular Accident; *UTI*  Urinary Tract Infection; *Abx*  Antibiotics

Table 2 shows details of infectious signs, symptoms and investigations. Indications for antibiotics consisting of urinary symptoms, fever or other infectious diagnoses were found in 93 cases (62%), thus leaving 57 patients (38%) in the asymptomatic cohort. In this asymptomatic subgroup, 68% (39 patients out of 57) were treated with antibiotics. Positivity rates of leukocyte, nitrite, and blood in the urinalysis were similar in both antibiotic-treated and untreated groups. However, we found higher numbers of patients with positive urine and blood cultures in the antibiotic-treated group. Culture positive UTI (positive culture with UTI symptoms or fever) was present in 19% and culture positive ASB consisted of 26%.

**Table 2:** Infectious symptoms and signs, investigations

	All patients (*N* = 150)	Given antibiotics (*N* = 129)	Not given antibiotics (*N* = 21)
Indications for antibiotics	93 (62%)	90 (70%)	3 (14%)
UTI symptoms	38 (25%)	36 (28%)	2 (10%)
Reported fever	29 (19%)	29 (23%)	0
ED-measured fever	7 (5%)	7 (5%)	0
Non-UTI diagnosis warranting antibiotic treatment	76 (51%)	75 (58%)	1 (5%)
Asymptomatic	57 (38%)	39 (30%)	18 (86%)
Urinalysis POCT performed	73 (49%)	62 (48%)	11 (52%)
Urinalysis by laboratory performed	134 (89%)	115 (89%)	19 (91%)
Urinalysis result			
Nitrite positive	47 (31%)	43 (33%)	4 (19%)
Leukocyte positive	147 (98%)	127 (98%)	20 (95%)
Blood positive	114 (76%)	101 (78%)	13 (62%)
Urine culture performed	127 (85%)	113 (88%)	14 (67%)
Positive	63 (42%)	61 (47%)	2 (10%)
Blood culture performed	96 (64%)	92 (71%)	4 (19%)
Positive	26 (17%)	26 (20%)	0
Culture positive UTI	28 (19%)	28 (22%)	0
Culture positive ASB	35 (26%)	33 (28%)	2 (13%)

*UTI*  Urinary Tract Infection; *POCT*  Point of Care Test; ED-measured fever = temperature 38.0 or higher; Asymptomatic = No UTI symptoms, fever, or other infectious diagnoses; Culture positive UTI = positive urine culture with UTI symptoms or fever; Culture positive ASB = positive urine culture without UTI symptoms or fever

Table 3 shows details of the bCAM positivity rate on the seventh day of admission. For the entire cohort, we observed no statistically significant differences, whether given antibiotics or not (RR 0.94 [0.41–2.16]). Subgroup analysis of the asymptomatic cohort also yielded a similar result with no statistical difference between antibiotic-treated and non-treated groups (RR 0.69 [0.22–2.15]). Sensitivity analysis was performed where we only used ED-measured fever, ignoring the patient-reported fever, to define the asymptomatic cohort, which also failed to show a statistical difference in seventh day bCAM positivity rate. Similarly, sensitivity analyses which looked at only culture positive UTI or ASB patients failed to show any significant difference in the delirium rate. On the seventh day of admission, there were no patients discharged yet, thus no patients were lost to follow up. All patients had bCAM assessments on the seventh day.

**Table 3:** bCAM positivity rate on the 7th day of admission

	Antibiotic	No antibiotic	RR [95% CI]
Entire cohort (*N* = 150)	22% (29/129)	24% (5/21)	0.94 [0.41–2.16]
Asymptomatic cohort (*N* = 57)	15% (6/39)	22% (4/18)	0.69 [0.22–2.15]
Modified asymptomatic cohort (*N* = 60)	19% (8/42)	22% (4/18)	0.86 [0.30–2.49]
Culture positive UTI (*N* = 28)	21% (6/28)	N/A	N/A
Culture positive ASB (*N* = 35)	27% (9/33)	50% (1/2)	0.55 [0.12–2.43]

*bCAM* brief confusion assessment method; Asymptomatic = no UTI symptoms, reported or ED-measured fever, or other infectious diagnoses; Modified asymptomatic = no UTI symptoms, ED-measured fever, or other infectious diagnoses; Culture positive UTI = positive urine culture with UTI symptoms or fever; Culture positive ASB = positive urine culture without UTI symptoms or fever; *N/A* not applicable

Mortality, ICU admission, *C. difficile* or institutionalization rates within 30 days of admission were similar in both the antibiotic-treated and non-antibiotic groups (Table 4).

**Table 4:** Number of patients with adverse outcomes (within 30 days of admission)

	Entire cohort (*N* = 150)			Asymptomatic cohort (*N* = 57)		
	Antibiotic (*N* = 129)	No antibiotic (*N* = 21)	RR [95% CI]	Antibiotic (*N* = 39)	No antibiotic (*N* = 18)	RR [95% CI]
Mortality	29 (22%)	4 (19%)	1.18 [0.46–3.02]	7 (18%)	4 (22%)	0.81 [0.27–2.41]
*C. difficile*	2 (2%)	0	N/A	0	0	N/A
ICU transfer	5 (4%)	0	N/A	0	0	N/A
ALC designation	74 (57%)	10 (48%)	1.20 [0.75–1.93]	24 (62%)	8 (44%)	1.38 [0.78–2.46]

Asymptomatic = no UTI symptoms, fever, or other infectious diagnoses; *C. difficile Clostridium difficile*; *ICU*  Intensive Care Unit; *ALC*  Alternate Level of Care; *N/A*  Not Applicable

Kappa values for antibiotic treatment (κ = 1), bCAM positivity on the seventh day of admission (κ = 1), and presence of dysuria, urgency, frequency, fever (κ = 0.93, 0.85, 1, 0.94) were high, suggesting excellent agreement between data abstractors. Kappa values for living arrangement, admission service and whether ED or admission diagnoses warranted antibiotic treatment were modest to good at 0.74, 0.65, and 0.64 each.

## Discussion

We conducted a health record review of older adults admitted to hospital who had both evidence of delirium and a positive urinalysis at or near the time of admission. In this population, a large majority of the patients were treated with antibiotics. More than one third of the patients were asymptomatic other the inclusion criteria of delirium and positive urinalysis. Two thirds of this asymptomatic subgroup was still given antibiotic treatment. These results suggest that, in this inpatient setting, treatment for UTI is common in older adults with delirium even in the absence of urinary symptoms.

The tendency to treat asymptomatic bacteriuria in patients with delirium is consistent with findings of the recent Canadian survey of physicians by Laguë et al. [3] The authors asked the respondents what the goals of antibiotic therapy were when treating bacteriuria in patients with delirium. “Infection treatment” was indicated by 83% and to “reduce the duration of delirium,” by another 65%. These answers suggest that physicians are, at least partially, convinced that bacteriuria found in patients with delirium could constitute an infection and such treatment could hasten resolution of delirium. The study also found, “pressure from family or colleagues” as another reason for such treatment. In our study, we noted a higher antibiotic treatment rate among the less independent population (retirement home and long-term care). Ability to communicate their symptoms and increased number of comorbidities may be factors in antibiotic treatment.

We did not observe a difference in delirium resolution on the seventh day of admission between those who were treated with antibiotics compared to those given no antibiotics. However, it is difficult to draw a conclusion given the obvious difference in the two comparison groups in terms of number of asymptomatic patients: patients who received antibiotics often had non-UTI indications for antibiotic treatment such as pneumonia or sepsis, whereas those who did not receive antibiotics were largely asymptomatic. In the subgroup analysis of the asymptomatic cohort, we again failed to see evidence that antibiotic treatment influenced the delirium rate on the seventh day of admission. However, the small number of patients, especially in the non-antibiotic-treated group makes the point prevalence imprecise with a large confidence interval, thus making it difficult to draw any firm conclusion. We also did not see any evidence that antibiotic treatment in this population was associated with rates of mortality, *C. difficile*, ICU transfer or ALC.

In this study, many older adults admitted with delirium and positive urinalysis had non-UTI diagnoses requiring antibiotics. UTI symptoms were present only in 25% of the population whereas twice as many patients had non-UTI infectious diagnoses. In fact, culture positive UTI was only about 20% of this population. We also found many potential alternative explanations for delirium such as fractures (pain), hyper/hyponatremia, hypercalcemia, hypothyroidism, seizure and stroke to name a few (Appendix 1). Thus, practitioners should be on the lookout for diagnoses other than UTI when faced with this population, in order to avoid availability and anchoring biases, where a positive urinalysis will stop practitioners from searching for further clinical data and prematurely conclude that the patient has a UTI.

In terms of the delirious but otherwise asymptomatic cohort, we still do not have a firm conclusion whether antibiotic treatment would improve delirium resolution. However, our study suggests that such treatment does not make a large difference as a large majority – nearly 4 out of 5 patients – would have resolution of delirium on the seventh day of admission, whether given antibiotics or not. These findings cast further doubt on whether a search for UTI in older adults with delirium but without urinary symptoms is beneficial. Given the high rates of ASB in older adults, investigating for UTI in older adults with delirium but without specific genitourinary UTI symptoms may only risk treating asymptomatic bacteriuria for which multiple studies have failed to prove benefits [9, 24, 25].

Our study did not show any difference in adverse outcomes such as 30-day mortality between antibiotic-treated cohort and non-treated cohort, in contrast to Pinnell’s health record review, which showed an increased 30-day and 6-months mortality [5]. Dasgupta et al. also showed worse functional outcomes including death and institutionalization when “asymptomatic UTI” was treated with antibiotics in the inpatient setting [26]. This may be due to a larger sample size in Pinnell’s study which had 499 patients vs. 150 patients in our study. We also note that Dasgupta’s study had 92 asymptomatic patients compared to 57 in our study. Additionally, in Pinnell’s study, the patient population was different from our study (undifferentiated confusion in ED vs. screened for delirium in inpatient units) although both studies are from the same institution.

Previous studies have attempted to establish a link between UTI/ASB and delirium, but often employed inconsistent definitions of UTI or used delirium itself as criteria for UTI, obscuring the results. Our study took on a slightly different and more practical question: whether the common practice of antibiotic treatment of older adults with delirium, when faced with a positive urinalysis, actually produces the intended result. To our knowledge, this is the first study that compared antibiotic treatment directly to delirium resolution in this population using a validated delirium screening tool. We also employed a strict case definition of asymptomatic patients and had no loss to follow up. We described this population in detail, provided the proportion of the asymptomatic cohort, and presented further equipoise of the antibiotic treatment in this asymptomatic group, which will enable future research.

Our study is limited by the nature of retrospective health record reviews. We are only able to report on documented signs and symptoms. Some patients may have had urinary symptoms which were not elicited or not documented in the health record. Similarly, the chronicity of the symptoms was not always clear in the documentation. Often no actual temperature was attached in the documentation regarding patient-reported fever. However, sensitivity analysis using only ED-measured fever yielded the same result (no significant difference in seventh day delirium rate) compared to using both ED-measured and patient reported fever. We also relied on routine nursing assessment for bCAM. However, bCAM instrument was validated in the context of non-physician use as mentioned previously. Due to heterogeneity of the entire cohort and small sample size of asymptomatic cohort and non-antibiotic treated cohort, we cannot make firm conclusions about the association between antibiotic treatment and delirium resolution. This study was conducted on the general medical inpatient units of a single academic tertiary care system, and our result may not be generalizable to other settings.

We agree with other researchers that a randomized control trial is necessary to further examine whether antibiotic treatment in older adults with delirium and positive urinalysis has an impact on delirium resolution, [4, 15] especially among those who have no other symptoms or indications for antibiotics.

## Conclusion

In this health record review of older adults admitted with delirium and positive urinalysis, we found that a majority of patients (86%) were treated with antibiotics, including 68% of patients with no urinary symptoms, fever, or other infectious diagnoses. We failed to find evidence that antibiotic treatment in this population was associated with faster delirium resolution on the seventh day of admission, or 30-day adverse outcomes such as mortality or ICU admission.

## Supplementary Information

**Additional file 1.**

## Data Availability

The datasets used and analysed during the current study are available from the corresponding author pending confirmation from the Ottawa Health Science Network Research Ethics Board.
