# Supplementary material for: Effect of inpatient antibiotic treatment among older adults with delirium found with a positive urinalysis: a health record review
Source: BMC Geriatr. 2022 Nov 29;22:916. doi: 10.1186/s12877-022-03549-8 (PMC9706880; doi:10.1186/s12877-022-03549-8)
Supplement: Supplementary file 1 — Additional file 1. [file 12877_2022_3549_MOESM1_ESM.docx]

**Appendix 1. List of diagnoses encountered in this health record review.** (They are reported as found in the records, other than acronyms which are spelled out.)

| **Infectious diagnoses potentially requiring antibiotic treatment** | **Diagnoses that do not require antibiotic treatment** |
| --- | --- |
| Bacteremia  *Clostridiodes difficile*  Cellulitis  Cerebellitis  Central nervous system infection  Dental infection  Encephalitis  Endocarditis  Fever  Gastrointestinal infection  Meningitis  Pneumonia  Prostatitis  Spontaneous bacterial peritonitis  Sepsis  Septic shock  Sinusitis  Systemic inflammatory response syndrome  Urosepsis  Urinary tract infection  Wound infection | Abdominal distention  Abdominal pain  Acute coronary syndrome  Acute decline  Acute kidney injury  Acute renal injury  Agitation  Altered loss of consciousness  Anemia  Aspiration  Aspiration pneumonitis  Atrial fibrillation  Back pain  Behavioural and psychological symptoms of dementia  Bladder obstruction  Chronic obstructive pulmonary disease exacerbation  Cirrhosis  Compression fracture  Confusion  Congestive heart failure  Congestive heart failure exacerbation  Constipation  Coronavirus disease-19  Delirium  Delusions  Dementia  Dementia progression  Diarrhea  Expressive aphasia  Failure to cope  Fall  Fracture  Functional decline  Generalized tonic-clonic seizure  Head injury  Hepatic cirrhosis  Hip fracture  Hypercalcemia  Hypercapnic respiratory failure  Hypernatremia  Hyperosmolar hyperglycemic syndrome  Hypertension  Hypokalemia  Hyponatremia  Hypotension  Hypothermia  Hypothyroidism  Hypoxia  Mallory-Weiss tear  Mechanical fall  Metastatic cancer  Mixed dementia  MSK pain  Nausea/Vomiting  Non-ST elevation myocardial infarction  Nonalcoholic steatohepatitis cirrhosis  Parotitis  Pleural effusion  Polypharmacy  Progressive decline  Progressive dementia  Progressive leg edema  Prostate mass  Psychosis  Rectal bleeding  Rectal pain  Recurrent falls  Renal failure  Rhabdomyolysis  Seizure  Seizures  Shortness of breath  Stasis dermatitis  Status epilepticus  Stroke  Syncope  Traumatic brain injury  Upper gastrointestinal bleed  Urinary retention  Upper respiratory tract infection  Vascular dementia  Violent behaviour  Viral meningitis/encephalitis  Viral respiratory illness  Viral upper respiratory tract infection  Weakness |
